# Acute Cannabigerol Administration Lowers Blood Pressure in Mice

**DOI:** 10.3389/fphys.2022.871962

**Published:** 2022-05-09

**Authors:** Victoria L. Vernail, Sarah S. Bingaman, Yuval Silberman, Wesley M. Raup-Konsavage, Kent E. Vrana, Amy C. Arnold

**Affiliations:** ^1^ Department of Neural and Behavioral Sciences, Penn State College of Medicine, Hershey, PA, United States; ^2^ Department of Pharmacology, Penn State College of Medicine, Hershey, PA, United States

**Keywords:** cardiovascular, mouse models, cannabinoids, adrenoreceptors, radiotelemetry

## Abstract

Cannabigerol is a cannabinoid compound synthesized by *Cannabis sativa*, which in its acid form acts as the substrate for both Δ^9^-tetraydrocannabinol and cannabidiol formation. Given its lack of psychoactive effects, emerging research has focused on cannabigerol as a potential therapeutic for health conditions including algesia, epilepsy, anxiety, and cancer. While cannabigerol can bind to classical cannabinoid receptors, it is also an agonist at α2-adrenoreceptors (α2AR) which, when activated, inhibit presynaptic norepinephrine release. This raises the possibility that cannabigerol could activate α2AR to reduce norepinephrine release to cardiovascular end organs to lower blood pressure. Despite this possibility, there are no reports examining cannabigerol cardiovascular effects. In this study, we tested the hypothesis that acute cannabigerol administration lowers blood pressure. Blood pressure was assessed *via* radiotelemetry at baseline and following intraperitoneal injection of cannabigerol (3.3 and 10 mg/kg) or vehicle administered in a randomized crossover design in male C57BL/6J mice. Acute cannabigerol significantly lowered mean blood pressure (−28 ± 2 mmHg with 10 mg/kg versus −12 ± 5 mmHg vehicle, respectively; *p* = 0.018), with no apparent dose responsiveness (−22 ± 2 mmHg with 3.3 mg/kg). The depressor effect of cannabigerol was lower in magnitude than the α2AR agonist guanfacine and was prevented by pretreatment with the α2AR antagonist atipamezole. These findings suggest that acute cannabigerol lowers blood pressure in phenotypically normal mice likely *via* an α2AR mechanism, which may be an important consideration for therapeutic cannabigerol administration.

## Introduction

Cannabinoids from *Cannabis sativa* are of growing interest due to their potential therapeutic benefits (Kovalchuk and Kovalchuk, 2020), and may represent an appealing alternative to current medications whose effectiveness is confounded by adverse side effects. In this regard, cannabis has been studied for treatment of chronic pain, cancer, epilepsy, anxiety, metabolic conditions, and several other diseases (Committee on the Health Effects of Marijuana et al., 2017). Compounds of particular interest include Δ^9^-tetraydrocannabinol (Δ^9^-THC), cannabidiol (CBD), and more recently cannabigerol (CBG). CBG, in its acid form, is the parent compound to cannabinoids such as Δ^9^-THC and CBD (Nachnani et al., 2021), and has similar properties to CBD including analgesia, neuroprotective, anticancer, anti-inflammatory, and antibacterial effects. In contrast to Δ^9^-THC, the main psychoactive component of cannabis which induces euphoria, both CBD and CBG lack psychoactive properties. There are other notable differences between these cannabinoids, including their binding at the classical cannabinoid receptors, cannabinoid receptor type 1 (CB1R) and type 2 (CB2R). While Δ^9^-THC is an agonist at CB2R and partial agonist at CB1R, CBD is an inverse agonist/antagonist at these receptors. CBG binds as a weak/partial agonist to CB1R and CB2R, but with lower affinity than Δ^9^-THC (Rosenthaler et al., 2014; Nachnani et al., 2021).

In addition to cannabinoid receptors, CBG appears to be a potent agonist with nanomolar affinity at α2-adrenoreceptors (α2AR) in peripheral tissues and the central nervous system (Cascio et al., 2010; Cathel et al., 2014). α2AR are presynaptic autoreceptors found both peripherally and centrally, which inhibit norepinephrine release to reduce sympathetic nervous system activity. Importantly, reduced norepinephrine release onto cardiovascular end organs, such as the heart and vasculature, can reduce vasoconstriction, heart rate, and cardiac contractility to lower blood pressure (Giovannitti et al., 2015). This raises the possibility that CBG may activate α2AR to lower blood pressure, which could have significant implications for potential therapeutic properties of this compound. Despite evidence for α2AR activation, the potential blood pressure effects of CBG remain unknown. Therefore, in this study, we tested the hypothesis that acute CBG administration can reduce blood pressure *via* an α2AR mechanism in conscious mice using a gold standard real-time radiotelemetry approach.

## Materials and Methods

### Approvals

All procedures were approved by the Institutional Animal Care and Use Committee at the Penn State College of Medicine and conformed to the NIH Guide for the Care and Use of Laboratory Animals.

### Animal Models

Male C57BL/6J mice (Jackson Laboratory, Bar Harbor, ME, United States) were used in these studies. Mice were fed a standard chow diet (Teklad 2018; Envigo, Indianapolis, IN, United States) and normal tap water *ad libitum* and housed on a 12-h light-dark schedule in a room with controlled humidity and temperature (∼23 °C). At approximately 8 weeks of age, mice were implanted with radiotelemetry probes (HD-X10, Data Sciences International, St. Paul, MN, United States), with the tip of the probe advanced *via* the carotid artery into the aortic arch, which allows for continuous measurement of blood pressure, heart rate, and locomotor activity in conscious freely moving animals. Following surgery, mice were individually housed and allowed to recover for at least 10 days prior to experiments.

### Acute CBG Administration

Baseline blood pressure, heart rate, and locomotor activity were recorded in conscious mice for 20 min prior to drug treatment. Mice then received a single intraperitoneal injection of CBG (3.3 and 10 mg/kg) or vehicle (1:1:18 DMSO: Tween80: Saline). Treatments were given in a randomized order, with at least one-week washout, in a crossover design. These doses and route of administration of CBG were chosen based on previous studies showing neuroprotective, anti-inflammatory, anti-nociceptive, and antiemetic effects in rodents (Rock et al., 2011; Borrelli et al., 2013; Valdeolivas et al., 2015; Zagzoog et al., 2020). Changes in blood pressure, heart rate, and locomotor activity from baseline were assessed for 3 hours post-injection, to capture the known time course for peak plasma concentrations of CBG following intraperitoneal injection in rodents (Deiana et al., 2012). Data were averaged into 5-min bins across the recording period to determine peak changes. Six mice were implanted with radiotelemetry probes for this experiment. The signal was lost in one probe mid-way through the study due to clots in the sensing catheter and, therefore, only five mice are included in the 3.3 mg/kg CBG dose. Animals were acclimated to intraperitoneal injections for 5 days prior to experiments to attempt to minimize confounding effects of handling stress.

### Potential α2AR Mechanisms Involved in CBG Depressor Effects

To determine if α2AR mechanisms are involved in the depressor effects of CBG, a second experiment was conducted using the same crossover study design in which a separate cohort of mice (*n* = 5) received intraperitoneal injection of CBG (10 mg/kg), the α2AR agonist guanfacine (1 mg/kg), or the α2AR antagonist atipamezole (3 mg/kg) + CBG (10 mg/kg). Atipamezole was given 10 min prior to CBG administration. The doses and time course for guanfacine and atipamezole were selected based on prior literature showing cardiovascular effectiveness of these drugs in mice (Janssen et al., 2004; Ying et al., 2014). The dose of CBG was selected as it produced significant blood pressure lowering effects in the first experiment following acute administration. To ensure our findings reflect pharmacological antagonism of CBG rather than additive effects of the combined drugs, we performed a third experiment in which a separate cohort of mice (*n* = 3) received intraperitoneal injection of atipamezole (3 mg/kg) or atipamezole (3 mg/kg) plus CBG (10 mg/kg; given at 10 min after atipamezole administration). Given the time course for CBG depressor effects in the first experiment, data were recorded for 2 hours post-drug administration and averaged in 5-min bins for these second and third experiments.

### Statistical Analysis

Data are presented as mean ± SEM and were analyzed by GraphPad Prism (Version 9.3.0). For experiments examining acute CBG versus vehicle administration, given the missing data within one dose, changes in blood pressure and heart rate were assessed *via* mixed effects models with post hoc Šídák’s multiple comparisons to account for the repeated measures and to assess main effects of drug, timepoint (baseline vs. post-drug), and their interaction. Locomotor activity was analyzed as an area under the curve (AUC) to summarize changes across the entire treatment period, and analyzed across groups *via* a mixed effects model to examine treatment effect. The time for peak changes in blood pressure following CBG and dose responsiveness for peak change in mean blood pressure were analyzed *via* paired *t*-test. For experiments examining potential α2AR mechanisms involved in CBG depressor effects, changes in blood pressure and heart rate following either CBG versus guanfacine, CBG versus atipamezole + CBG, or atipamezole versus atipamezole + CBG were assessed *via* two-way repeated measures ANOVA to assess main effects of drug, time, and their interaction with post hoc Šídák’s multiple comparisons. A *p*-value of <0.05 was considered statistically significant.

## Results

### Acute CBG Lowers Blood Pressure, but Does Not Alter Heart Rate or Locomotor Activity

**FIGURE 1:**
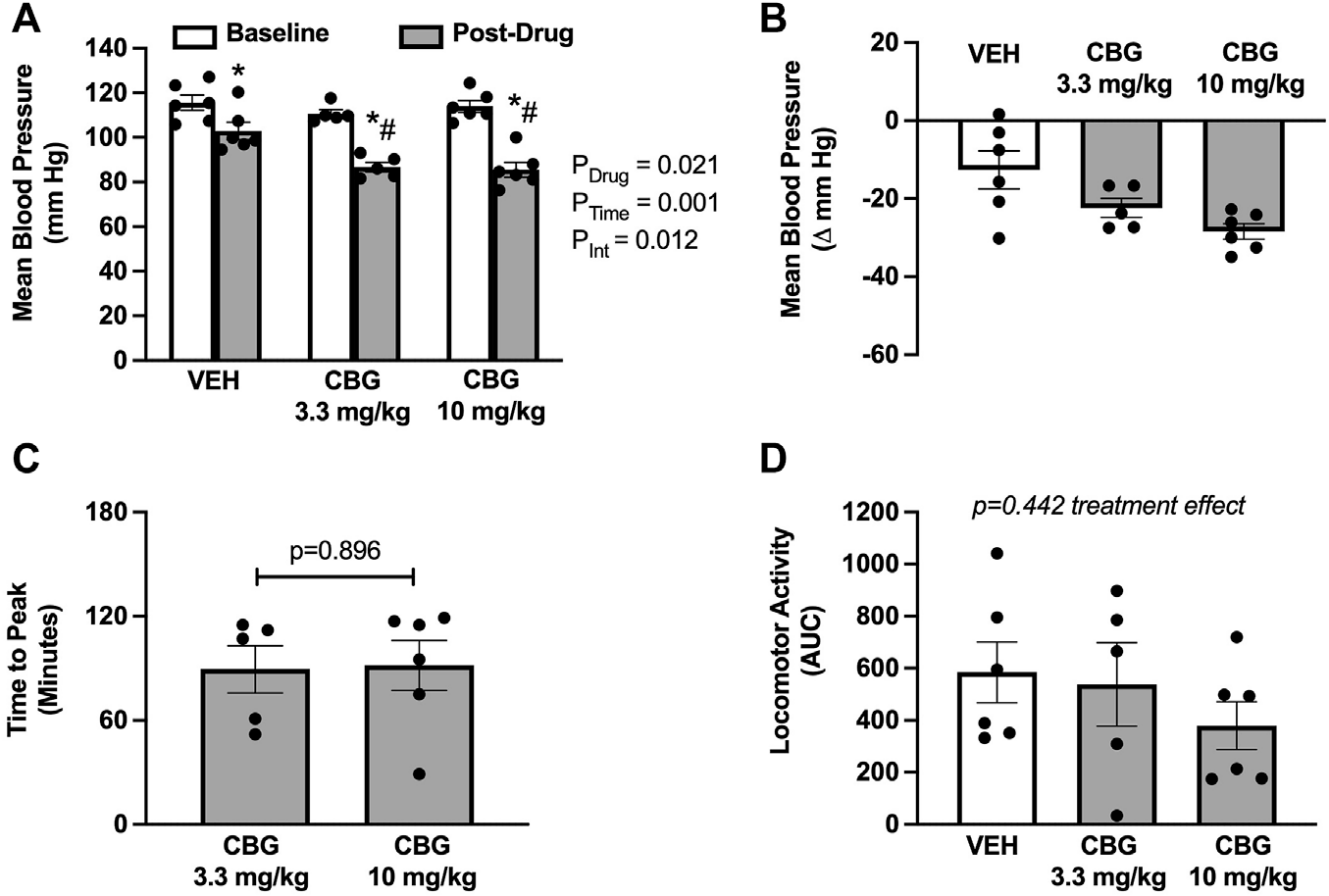
Acute cannabigerol (CBG) administration decreases mean blood pressure. **(A)** CBG significantly lowered mean blood pressure when compared with vehicle (VEH) administration (P_Drug_ = 0.021, P_Time_<0.001, P_Int_ = 0.019; mixed effects model; *p < 0.05 versus baseline and # *p* < 0.05 versus vehicle following post hoc Šídák’s multiple comparisons test). **(B)** The magnitude of depressor effect of CBG was greater than VEH when shown as the peak change from baseline (−28 ± 2 at 10 mg/kg *vs*. −22 ± 2 at 3 mg/kg *vs*. −12 ± 5 mmHg vehicle). **(C)** The peak blood pressure lowering effect of CBG occurred on average at ∼90 min for both 3.3 and 10 mg/kg doses (*p* = 0.896 paired *t*-test). **(D)** There were no differences in the area under the curve (AUC) for locomotor activity over the study period across treatments [f (2,9) = 0.895; *p* = 0.442 mixed effects model]. N = 5–6/group.

Acute CBG administration elicited a significant decrease in mean blood pressure compared with vehicle (Figure 1A). There was no apparent dose responsiveness for the CBG doses used in this study (Figure 1B; Δ from baseline: −22 ± 2 at 3.3 mg/kg versus −28 ± 2 mmHg at 10 mg/kg). On average, the peak decrease in mean blood pressure elicited by CBG was observed at 90-min post-administration, with no difference between doses (Figure 1C). CBG also significantly lowered systolic and diastolic blood pressure compared with vehicle (Table 1), again with no dose responsiveness. There were no differences in heart rate responses following CBG versus vehicle administration (Table 1). There were also no differences in locomotor activity over the study period across treatment groups (Figure 1D).

**TABLE 1:** Blood pressure and heart rate following acute CBG administration.

Parameter, units	Vehicle	CBG	CBG	P_Drug_	P_Time_	P_Int_
		3.3 mg/kg	10 mg/kg			
**SBP, mmHg**						
Baseline	132 ± 4	128 ± 2	131 ± 3	0.029	0.001	0.018
Post-Drug	120 ± 5*	103 ± 3*	99 ± 4*			
**DBP, mmHg**						
Baseline	106 ± 6	104 ± 3	106 ± 3	0.173	0.001	0.049
Post-Drug	89 ± 6*	71 ± 3*	73 ± 4*			
**HR, bpm**						
Baseline	583 ± 27	518 ± 14	481 ± 11	0.003	0.004	0.502
Post-Drug	527 ± 22	439 ± 23*	450 ± 25			

CBG, cannabigerol; SBP, systolic blood pressure; DBP, diastolic blood pressure; HR, heart rate.

Data are mean ± SEM and were analyzed by mixed effects models to assess for main effects of vehicle versus CBG treatment (P_Drug_), baseline versus post-drug timepoint (P_Time_), and their interaction (P_Int_).

*p < 0.05 versus baseline following post hoc Šídák’s multiple comparisons test.

### Acute CBG Lowers Blood Pressure *via* an α2AR Mechanism

**FIGURE 2:**
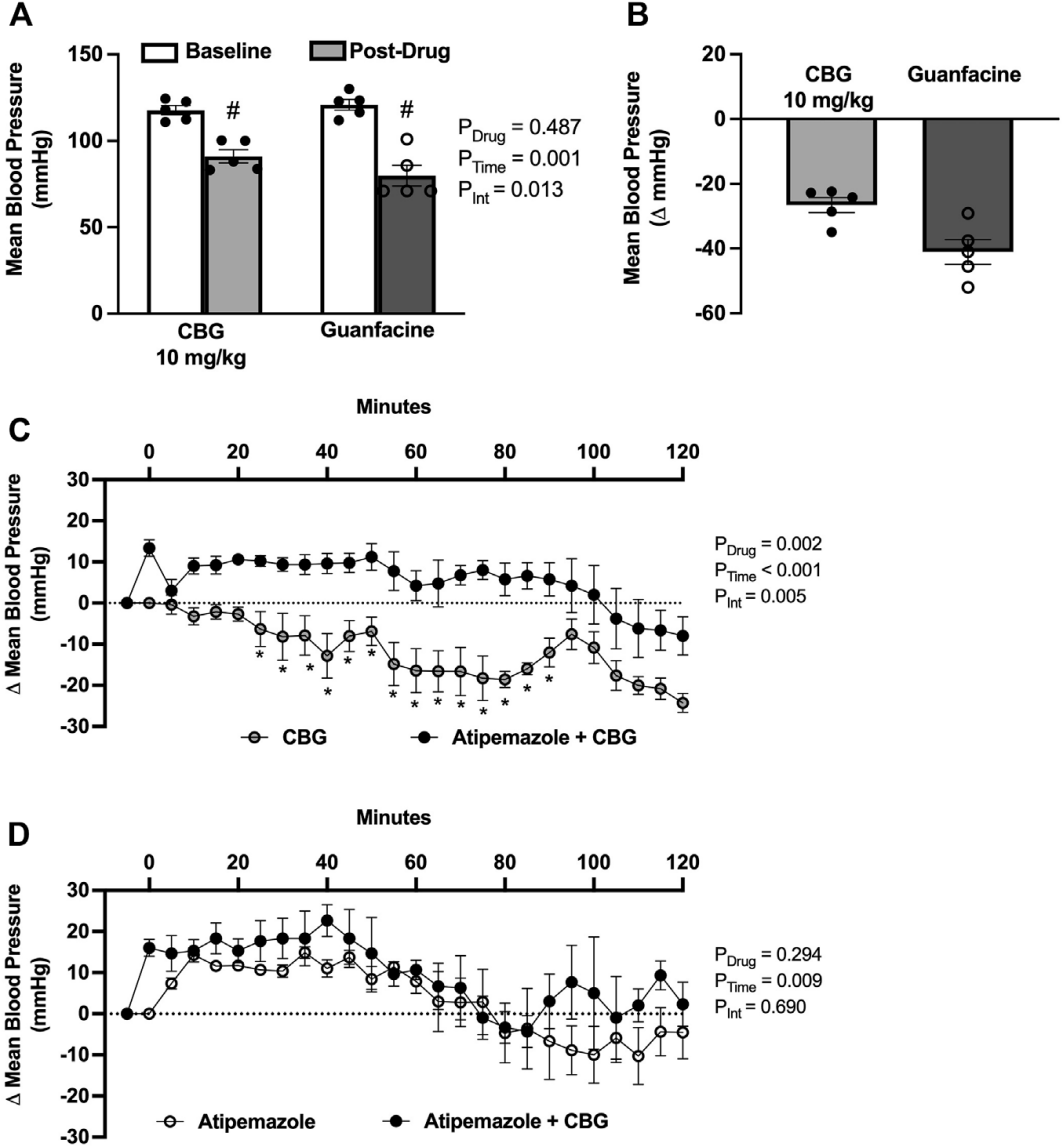
Acute cannabigerol (CBG) lowers blood pressured *via* an alpha2-adrenoreceptor (α2AR) mechanism. **(A)** CBG (10 mg/kg, i.p.) lowered mean blood pressure to a lesser extent than guanfacine (1 mg/kg, i.p.) administration (P_Drug_ = 0.487, P_Time_ = 0.001, P_Int_ = 0.013; two-way repeated measures ANOVA; # *p* < 0.01 versus baseline following post hoc Šídák’s multiple comparisons test; N = 5/group). **(B)** The magnitude of depressor effect of CBG was less than guanfacine when shown as the peak change from baseline (−27 ± 2 *vs*. −41 ± 4 mmHg, respectively). **(C)** Pretreatment with the α2AR antagonist atipamezole (3 mg/kg, i.p.) prevented CBG (10 mg/kg, i.p.) blood pressure lowering effects (P_Drug_ = 0.001, P_Time_<0.001, P_Int_ = 0.001; two-way repeated measures ANOVA; *p < 0.05 versus atipamezole + CBG following post hoc Šídák’s multiple comparisons tests; N = 5/group). **(D)** There were no significant differences in the blood pressure effects of atipamezole versus atipamezole plus CBG (P_Drug_ = 0.294, P_Time_<0.001, P_Int_ = 0.690; two-way repeated measures ANOVA; N = 3/group).

To determine if the acute blood pressure lowering effects of CBG are mediated *via* an α2AR mechanism, we first compared the magnitude of depressor response to a known α2AR agonist, guanfacine. We found that both CBG and guanfacine lowered mean blood pressure from baseline, with the peak decrease being smaller for CBG (Figures 2A,B). We next determined if the blood pressure lowering effects of CBG could be prevented by pretreatment with atipamezole, an α2AR antagonist. Atipamezole alone caused an ∼13 mmHg increase in mean blood pressure, reflecting disinhibition of norepinephrine release *via* α2AR. Importantly, atipamezole prevented the drop in mean blood pressure elicited by acute CBG administration (Figure 2C). Mean blood pressure responses to atipamezole versus atipamezole plus CBG were similar (Figure 2D).

## Discussion

The cannabinoid system is involved in several aspects of cardiovascular regulation including control of blood vessel tone, cardiac contractility, blood pressure, and vascular inflammation (Pacher et al., 2005). CBG is a less studied phytocannabinoid with evidence emerging for many potential beneficial effects; however, its impact on the cardiovascular system is unknown. We found that acute CBG administration elicits a significant decrease in blood pressure in phenotypically normal male mice, without altering heart rate or locomotor activity. Additionally, our data suggest that the blood pressure lowering effects of CBG are mediated through an α2AR mechanism. Overall, the present study provides new mechanistic insight into the cardiovascular effects of CBG in the context of normal blood pressure.

Currently, little is known about the effects of CBG on the cardiovascular system, with no reports related to blood pressure. To our knowledge, only one study has examined cardiovascular-related effects of CBG and showed inhibition of platelet aggregation induced by adrenaline in human platelets suggesting anti-thrombotic activity (Formukong et al., 2011). We found that acute CBG lowered mean blood pressure in mice by ∼28 mmHg (compared with ∼12 mmHg for vehicle treatment), without effects on heart rate. The lack of a compensatory heart rate increase in response to the decrease in blood pressure with CBG may indicate impairment of the arterial baroreceptor reflex, which is a homeostatic mechanism to correct for changes in blood pressure. However, this impairment remains to be tested. We did not observe evidence of dose-responsiveness for CBG; however, only two doses were fully examined in this study and future studies may need to include additional doses of CBG. The 10 mg/kg dose of CBG has been widely shown as effective in rodents models of neurodegenerative diseases and inflammatory bowel disease (Rock et al., 2011; Borrelli et al., 2013). An intermediate dose of 5.6 mg/kg was tested in three mice in the present study, but again, showed no evidence for dose responsiveness in terms of ability to lower mean blood pressure (−28 ± 4 mmHg). Higher doses were not tested as 10 mg/kg of CBG decreased blood pressure substantially, and it is likely a floor effect would be observed. Our finding for lack of dose responsiveness is consistent with a recent report showing variability in effective concentrations for CBG in terms of analgesic and anti-inflammatory effects in mice (Kogan et al., 2021).

The peak effects of CBG occurred on average at 90 min post administration, which is within the time course previously reported for peak plasma concentrations of CBG in rodents (Deiana et al., 2012). This prolonged time course for effects on blood pressure may suggest activation of central neurocircuits, as direct vascular effects of vasoactive agents typically occur within a shorter time frame [e.g., seconds-minutes; (White et al., 1999)]. Additionally, cannabinoids such as Δ^9^-THC, as well as synthetic cannabinoid receptor agonists, are known to decrease psychomotor activity resulting in sedation (Bosier et al., 2010; Boggs et al., 2018), which could limit their use for therapeutic treatment. Our results show that CBG did not acutely alter locomotor activity, which is consistent with a lack of psychomotor and sedative effects to confound blood pressure changes. As mice were studied during the light cycle, however, it is unclear if CBG would impact blood pressure and locomotor activity differently during the more active dark cycle.

We further provide evidence that depressor effects of CBG are mediated by an α2AR mechanism. Agonists of α2AR, such as guanfacine and clonidine, act centrally with peak plasma concentrations within 1–4 h (Dollery and Davies, 1980; van Zwieten et al., 1984). These drugs are of therapeutic interest due to their antihypertensive and analgesic properties, but are often limited by sedation. Previous studies have shown that CBG can activate α2AR, and that these receptors are involved in effects on hyperphagia and peripheral tissue contraction (Cascio et al., 2010; Brierley et al., 2016). In support of this, we show that CBG depressor effects are prevented by pretreatment with the α2AR antagonist atipamezole. We further show that blood pressure effects of atipamezole are similar to that of atipamezole plus CBG, supporting pharmacological α2AR antagonism rather than additive effects of the drug combination. Of interest, CBG lowered blood pressure to a lesser extent than the α2AR agonist guanfacine. It is known that guanfacine has high selectivity for the α2_a_ receptor subtype in brain and reduces blood pressure less than clonidine. Similar to our findings, CBG was found to have less potency and efficacy than the α2AR agonist dexmedetomidine at inhibiting electrically-evoked vas deferens contraction (Cascio et al., 2010). Thus, it is possible that CBG is a less potent α2AR agonist, engages a different α2AR subtype, or can act on peripheral versus central α2AR for cardiovascular effects. Unfortunately, pharmacological probes and animal models to dissect the importance of peripheral versus central α2AR to CBG effects are not currently available.

To date, research on the effects of cannabinoid compounds on cardiovascular function is conflicting, with some studies showing cardioprotection and others showing adverse cardiovascular outcomes (Mittleman et al., 2001; Mukamal et al., 2008; Wolff et al., 2011; Abuhasira et al., 2021). These disparate findings may reflect differences in cannabinoids and the models used, mode of administration, or effects on cannabinoid versus other receptors in the periphery and central nervous system. Our finding that CBG lowers blood pressure is consistent with reports that: synthetic cannabinoids including Δ^9^-THC may decrease blood pressure (Jones, 2002); cannabis users have increased risk of orthostatic hypotension (Mathew et al., 1992); CBD causes vasorelaxation in human mesenteric arteries (Stanley et al., 2015) and lowers blood pressure in hypertensive rats (Baranowska-Kuczko et al., 2020); and the sympathetic nervous appears to play a role in the cardiovascular depression effects of endocannabinoids (Niederhoffer et al., 2003; Dean, 2011). Despite this literature for other cannabinoids, very little is known about adverse effects or medication interactions for CBG, including potential effects on the cardiovascular system. Our findings may suggest caution against use of CBG in healthy individuals due to the potential for hypotension but may provide a new therapeutic approach to lower blood pressure in the context of hypertension.

Overall, these findings add to the growing literature regarding the role of cannabinoids in blood pressure regulation. Additional research on CBG is needed to define the precise molecular mechanisms and sites of action, effects of more chronic administration, and potential for therapeutic use to lower blood pressure in models of hypertension. Further, this study only included male mice and thus sex differences were not explored. Females tend to have lower resting blood pressure (Sandberg and Ji, 2012; Joyner et al., 2016) and are more sensitive to α2AR agonism (Mitrovic et al., 2003), and thus may exhibit greater depressor responses to CBG. Our findings suggest that acute CBG lowers blood pressure in phenotypically normal mice likely *via* an α2AR mechanism, which may be an important consideration for therapeutic CBG administration.

## Data Availability

The original contributions presented in the study are included in the article/Supplementary Material, further inquiries can be directed to the corresponding author.
